# The adequacy of encephalitis surveillance for emerging infectious diseases in Australia

**DOI:** 10.1186/1742-4690-9-S1-O9

**Published:** 2012-05-25

**Authors:** David N Durrheim, Clare Huppatz, Beverley Paterson

**Affiliations:** 1 University of Newcastle, Wallsend, Australia; 2 Hunter Medical Research Institute, University of Newcastle, Wallsend, Australia

## Introduction

The acute encephalitis syndrome has heralded the emergence of multiple virulent pathogens in Australia, including Murray Valley encephalitis, and recently Hendra virus and Australian bat lyssavirus. In Australia, the encephalitis syndrome in humans is not notifiable. The burden of Australian encephalitis hospitalisations and deaths was not known; encephalitis aetiology and trends had not been investigated at population level; and the adequacy of neurological review in hospitalised encephalitis cases to exclude emerging pathogens had not been investigated.

## Materials and methods

A series of studies was conducted to better understand the status of encephalitis surveillance in Australia. Firstly, Australian Bureau of Statistics mortality and population data for the period 1979–2006 were obtained and cause of death data were extracted using ICD-9 (1979–1998) and ICD-10 (1999–2006). Secondly, rates of hospitalisation for patients with encephalitis in Australia’s most populous state, New South Wales (NSW), from January 1990 through to December 2007 were reviewed, with encephalitis-associated hospital stays extracted using ICD-9-CM (1990–1998) and ICD-10-AM (1999–2007) from the NSW Department of Health Inpatient Statistics Collection library. Finally, a retrospective clinical audit was performed, of all adult encephalitis admissions between July 1998 and December 2007 to the three hospitals with adult neurological services in northern NSW. Case notes were examined for evidence of relevant history taking, clinical features, physical examination, laboratory and neuroradiology investigations, and outcomes.

## Results

Between 1979 and 2006 there were 1,118 encephalitis-associated deaths in Australia, with an average annual death rate of 2.3 per million population. The aetiology of 576 deaths was unknown and the proportion of deaths due to ‘unknown’ encephalitis increased from 47.0% between 1979 and 1992, to 57.2% from 1993 to 2006. Encephalitis was the primary discharge diagnosis for 5,926 hospital admissions in NSW with an average annual hospitalisation rate of 5.2/100,000 population. Toxoplasma encephalitis and subacute sclerosing panencephalitis showed notable declines. The proportion of patients hospitalised with encephalitis and no identified pathogen (69.8%, range 61.5%–78.7%) was stable during the study period. Amongst patients admitted with an encephalitis diagnosis in the NSW regional neurological hospital network, treating clinicians suspected a specific causative organism in 18.9% of cases and a cause was confirmed by laboratory testing in 12.1% of cases. However, only 14.9% were tested for flaviviruses and tests for specific locally occurring zoonotic encephalitis viruses were only conducted in 0.0-6.8% of cases.

## Conclusions

The non-notifiable status of human encephalitis in Australia and the high proportion of cases, including deaths, with no known aetiology may conceal emergence of novel pathogens. Unexplained encephalitis should be investigated, and encephalitis hospitalisations should be subject to statutory notification to facilitate prompt public health investigation and action as necessary. The utility of hospital sentinel surveillance, and standardised diagnostic and testing algorithm is currently being explored.

